# Multigene phylogeny, taxonomy and reclassification of *Hyaloperonospora* on *Cardamine*

**DOI:** 10.1007/s11557-013-0900-z

**Published:** 2013-04-14

**Authors:** Hermann Voglmayr, Young-Joon Choi, Hyeon-Dong Shin

**Affiliations:** 1 Department of Systematic and Evolutionary Botany, Faculty Center Biodiversity, University of Vienna, Rennweg 14, 1030 Wien, Austria; 2 Biodiversity and Climate Research Centre (BiK-F), Senckenberganlage 25, D-60325 Frankfurt (Main), Germany; 3 Division of Environmental Science and Ecological Engineering, Korea University, Seoul, 136-701 Korea; 4 Department of Biological Sciences, Institute of Ecology, Evolution and Diversity, Goethe University, Siesmayerstr. 70, D-60323 Frankfurt am Main, Germany

**Keywords:** *Dentaria*, Host range, Obligate parasites, *Peronospora*, *Peronosporaceae*

## Abstract

Based on sequence data from *cox1*, *cox2*, ITS and LSU rDNA, it is shown that at least six species of *Hyaloperonospora* occur on the genus *Cardamine*, most of which were commonly classified under *Peronospora dentariae*. Based on sequences from their type hosts, *Peronospora dentariae*, *Peronospora cardamines-laciniatae*, *Peronospora dentariae-macrophyllae*, *Peronospora malyi* and *Peronospora nasturtii-aquatici* are combined into *Hyaloperonospora*, and their circumscription is clarified. *Hyaloperonospora cardamines-enneaphyllos* is described as a new species from *Cardamine enneaphyllos*. The host range of *Hyaloperonospora nasturtii-aquatici*, described from *Nasturtium officinale*, is shown to extend to various *Cardamine* species. Host range of species is shown to be highly diagnostic, with no overlap in their host range, but species commonly cannot be distinguished by morphology alone. Both *cox1* and *cox2* are confirmed to be good markers for phylogenetic species delimitation of closely related *Hyaloperonospora* species on *Cardamine*.

## Introduction

Recent molecular phylogenetic studies showed that the genus *Hyaloperonospora*, established by Constantinescu and Fatehi (2002) for *Peronospora* species infecting *Brassicaceae*, *Capparaceae*, *Cistaceae*, *Limnanthaceae*, *Resedaceae* and *Zygophyllaceae*, comprises a high biodiversity, its species usually being highly host specific (e.g. Riethmüller et al. 2002; Choi et al. 2003; Voglmayr 2003; Göker et al. 2003, 2004, 2009a; Choi et al. 2011; Voglmayr and Göker 2011). Therefore, a narrow species circumscription as already advocated by Gäumann (1918, 1923) has been confirmed, disproving the widely applied concept of Yerkes and Shaw (1959), who classified all accessions from *Brassicaceae* under a single species, *H. parasitica* (see review in Voglmayr 2008).

Although in general the narrow species concept of Gäumann (1918, 1923) has been shown to be more appropriate, there are numerous problems in detail. Gäumann (1918, 1923) described numerous *Peronospora* species from *Brassicaceae* based on subtle morphological differences and on evidence of high host specificity obtained by cross-inoculation studies; however, as cross-inoculation studies could be performed only on a very limited number of hosts, most of his species were mainly based on host range in combination with often subtle differences in conidial size and shape. Therefore, he commonly classified accessions of various related host species under the same species if they were morphologically similar. As a result, he unfortunately did not select types for the species he described, but only a list of material examined, which especially becomes a problem if accessions from several hosts were classified under the same species. Recently, the extensive molecular phylogenetic study of Göker et al. (2009a) showed that several of his species were highly polyphyletic, as accessions from related hosts classified under the same species name by Gäumann (1918) are often not closely related, raising the problem of the correct naming of species. For this, nomenclatural and taxonomic decisions like lectotypification of heterogeneous entities are necessary. In addition, sequences have to be available for the species originating from the type host before appropriate reclassification can be achieved.

Five of these problematic taxa for which no nomenclatural decisions could yet be achieved due to reasons discussed above include *Peronospora dentariae*, *P. cardamines-laciniatae*, *P. dentariae-macrophyllae*, *P. malyi* and *P. nasturtii-aquatici*. *Peronospora dentariae* has already been described by Rabenhorst (1859) from an Italian collection from *Cardamine* (*Dentaria*) *heptaphylla*, a rather rare montane species confined to the Jura, south-western Alps and Apennines, but Gäumann (1918, 1923) also placed collections from various other *Cardamine* species in that species. *Peronospora cardamines-laciniatae*, *P. dentariae-macrophyllae* and *P. nasturtii-aquatici* have been described by Gäumann (1918), based on differences in conidial sizes. *Peronospora cardamines-laciniatae* was described for accessions from the North American *Cardamine laciniata*, *P. dentariae-macrophyllae* for accessions from the East Asian *Cardamine leucantha* (syn. *C. macrophylla* var. *dasyloba*), and *P. nasturtii-aquatici* for accessions from *Nasturtium officinale*. Based on his conidial measurements, Gäumann (1918) assumed that accessions from the European *Cardamine bulbifera* could also belong to *P. cardamines-laciniatae*, but later expressed strong doubts that they are conspecific (Gäumann 1923). However, Gustavsson (1959) found no significant differences in conidial size between accessions from various *Cardamine* species either classified as *P. dentariae* or *P. cardamines-lacinatae* and he considered them to be synonymous. Finally, Lindtner (1957) described a fifth species, *Peronospora malyi*, from *Cardamine graeca*, based on larger conidia and oospores.

The extensive investigation of Göker et al. (2009a) showed that *Hyaloperonospora* accessions from various *Cardamine* species were placed in five distinct subgroups, two of which were part of their clade 1, and three of their clade 6. However, as neither material from the type host of *Peronospora dentariae*, *C. heptaphylla*, nor from the type host of *P. cardamines-laciniatae*, *C. laciniata*, were available for study at that time, it remained unclear to which clades the names *P. dentariae* and *P. cardamines-laciniatae* should be applied. Therefore, the various *Hyaloperonospora* clades on *Cardamine* could not be properly classified. Due to these uncertainties, *P. nasturtii-aquatici* could also not be properly classified, as accessions from *Nasturtium officinale* were contained in a clade comprising accessions from various *Cardamine* species, e.g., *C. amara*, *C. hirsuta* and *C. pratensis*, which were all placed in the older *P. dentariae* by Gäumann (1918). No material from *Cardamine graeca*, the type host of *P. malyi*, was included in the investigation of Göker et al. (2009a), so the status of that species also remained unresolved.

To clarify these nomenclatural uncertainties, recent collections were obtained for the type hosts of all four species described from *Cardamine*. Four genes (ITS, LSU, *cox1*, *cox2*) were sequenced and analyzed for numerous accessions from *Cardamine* to evaluate the host ranges and species boundaries.

## Materials and methods

### Morphological analysis

Conidiophores and conidia were removed from the underneath of infected leaves, transferred to anhydrous lactic acid on a slide, carefully torn apart using forceps and needles, shortly heated using an alcohol burner and covered with a cover slip. Slides were examined and photographed using a Zeiss Axio Imager.A1 (Zeiss, Jena, Germany) microscope equipped with a Zeiss AxioCam ICc3 digital camera. Measurements are reported as maxima and minima in parentheses and the mean plus and minus the standard deviation of a number of measurements given in parentheses.

### Sample sources

Information on the samples used for morphological analysis, sequencing and phylogenetic analyses is given Table 1.

**Table 1:** Sources and GenBank accession numbers of *Hyaloperonospora* and *Perofascia* material used for molecular phylogenetic analyses. For institution codes of herbarium vouchers, see Thiers (2013); asterisks (*) denote sequences generated in the present study

					GenBank accession no.			
Taxon	Host	Geographic origins	Accession	Voucher	ITS	LSU	*cox1*	*cox2*
*Hyaloperonospora arabidopsidis*	*Arabidopsis thaliana*	Austria, Oberösterreich, St. Willibald	HV2091	WU	EU049236	EU054900	–	–
*H. barbareae*	*Barbarea vulgaris*	Austria, Tirol, Schattwald	MG1862	TUB 12260	AY531395	AY035499	–	–
*H. brassicae*	*Brassica napus*	Austria, Oberösterreich, St. Willibald	HV2163	WU	EU049248	EU054911	–	–
*H. camelinae*	*Camelina sativa*	Austria, Oberösterreich, St. Willibald	HV444	WU	AY531456	EU054841	–	–
*H. cardamines-laciniatae*	*Cardamine diphylla*	USA, Tennessee, Knoxville	HV-PA10	WU 32372	KC494994*	KC494994*	KC494918*	KC494953*
*H. cardamines-laciniatae*	*C.* cf. *diphylla*	USA, Tennessee, Great Smoky Mts Natl. Park	HV2.4.P.P.	WU 32373	KC494995*	KC494995*	KC494919*	KC494954*
*H. cardamines-laciniatae*	*C. laciniata*	USA, Maryland, Beltsville	HV2949	WU 32371	KC494996*	KC494996*	KC494920*	KC494955*
*H. cardaminopsidis*	*Arabidopsis arenosa*	Germany, Sachsen, Plattenthal	D23/7/97	TUB 12446	AY531435	EU054829	–	–
*H. cheiranthi*	*Erysimum cheirei*	Germany, Sachsen-Anhalt, Plossig	J3786/01	TUB 12419	AY531460	EU054849	–	–
*H. dentariae*	*Cardamine bulbifera*	Austria, Niederösterreich, Gießhübl	HV77	WU 22896	AY531399	EU054837	KC494921*	KC494956*
*H. dentariae*	*C. bulbifera*	Austria, Niederösterreich, Gießhübl	HV2029	WU 32375	KC494997*	KC494997*	–	KC494957*
*H. dentariae*	*C. bulbifera*	Austria, Niederösterreich, Mannersdorf/Leithageb.	HV2100	WU 32376	KC494998*	KC494998*	KC494922*	KC494958*
*H. dentariae*	*C. bulbifera*	Austria, Niederösterreich, Kaltenleutgeben	HV2106	WU 32377	KC494999*	KC494999*	KC494923*	KC494959*
*H. dentariae*	*C. bulbifera*	Austria, Niederösterreich, Tattendorf	HV2260	WU 32378	KC495000*	KC495000*	KC494924*	KC494960*
*H. dentariae*	*C. bulbifera*	Germany, Baden-Württemberg, Tübingen	MG2144	TUB 12303	EU049251	EU054913	–	–
*H. dentariae*	*C. heptaphylla*	Italy, Trentino, Storo, Val di Lorina	HV2927	WU 32379	KC495001*	KC495001*	KC494925*	KC494961*
*H. dentariae*	*C. heptaphylla*	Italy, Trentino, Storo, Val delle Communi	HV2928	WU 32380	KC495002*	KC495002*	KC494926*	KC494962*
*H. dentariae*	*C. heptaphylla*	Italy, Trentino, Val di Ampola	HV2930	WU 32381	KC495003*	KC495003*	KC494927*	KC494963*
*H. dentariae*	*C. heptaphylla*	Italy, Lombardia, Endine Gaiano, Valmaggiore	HV2932	WU 32382	KC495004*	KC495004*	KC494928*	KC494964*
*H. dentariae*	*C. heptaphylla*	Italy, Lombardia, Lecco, Canzo, Gajum	HV2935	WU 32383	KC495005*	KC495005*	KC494929*	KC494965*
*H. dentariae*	*C. heptaphylla*	Italy, Lombardia, Bellagio, Monte San Primo	HV2938	WU 32384	KC495006*	KC495006*	KC494930*	KC494966*
*H. dentariae*	*C. heptaphylla*	Italy, Lombardia, Valle Imagna, Valsecca	HV2941	WU 32385	KC495007*	KC495007*	KC494931*	KC494967*
*H. dentariae*	*C. impatiens*	Germany, Baden-Württemberg, Tübingen	HV855	WU 32386	KC495008*	KC495008*	-	KC494968*
*H. dentariae*	*C. impatiens*	Austria, Niederösterreich, Gießhübl	HV2174	WU 32387	–	–	–	KC494969*
*H. dentariae*	*C. impatiens*	Austria, Niederösterreich, Gießhübl	HV2289	WU 32388	KC495009*	KC495009*	KC494932*	KC494970*
*H. dentariae*	*C. impatiens*	Austria, Tirol, Steeg		GLM46902	EU049231	EU054896	–	–
*H. dentariae*	*C. impatiens*	Austria, Tirol, Schattwald	MG1840	TUB 12261	AY531400	AY035500	–	–
*H. dentariae*	*C. impatiens*	Germany, Baden-Württemberg, Tübingen	MG1939	TUB 12282	AY531397	AY272000	–	–
*H. dentariae*	*C. pentaphyllos*	Austria, Kärnten, Ferlach, Tscheppaschlucht	HV2334	WU 32389	KC495010*	KC495010*	KC494933*	KC494971*
*H. dentariae*	*C. pentaphyllos*	Italy, Lombardia, Endine Gaiano, Valmaggiore	HV2933	WU 32390	KC495011*	KC495011*	KC494934*	KC494972*
*H. dentariae*	*C. pentaphyllos*	Italy, Friuli, Val di Resia, Lischiazze	HV2944	WU 32391	KC495012*	KC495012*	KC494935*	KC494973*
*H. dentariae-macrophyllae*	*C. leucantha*	Korea, Chuncheon	SMK17273	KUS-F 17273	AY210990	KC495013*	KC494936*	KC494974*
*H. dentariae-macrophyllae*	*C. leucantha*	Korea, Gangneung	SMK17298	KUS-F 17298	AY210991	KC495014*	KC494937*	KC494975*
*H. dentariae-macrophyllae*	*C. leucantha*	Korea, Hongcheon	SMK17322	KUS-F 17322	AY210992	KC495015*	KC494938*	KC494976*
*H. dentariae-macrophyllae*	*C. leucantha*	Korea, Gangneung	SMK17539	KUS-F 17539	AY210993	KC495016*	KC494939*	KC494977*
*H. cardamines-enneaphyllos*	*C. enneaphyllos*	Austria, Niederösterreich, Gießhübl	HV2025	WU 32392	KC495017*	KC495017*	–	–
*H. cardamines-enneaphyllos*	*C. enneaphyllos*	Austria, Niederösterreich, Mannersdorf/Leithageb.	HV2099	WU 32393	KC495018*	KC495018*	KC494940*	KC494978*
*H. cardamines-enneaphyllos*	*C. enneaphyllos*	Austria, Niederösterreich, Gießhübl	HV2105	WU 32394	KC495019*	KC495019*	–	KC494979*
*H. cardamines-enneaphyllos*	*C. enneaphyllos*	Austria, Steiermark, Gröbming	HV2125	WU 32395	KC495020*	KC495020*	KC494941*	KC494980*
*H. cardamines- enneaphyllos*	*C. enneaphyllos*	Italy, Trentino, Storo, Val delle Communi	HV2929	WU 32396	KC495021*	KC495021*	KC494942*	KC494981*
*H. hesperidis*	*Hesperis matronalis*	Germany, Sachsen-Anhalt, Kloster Gröningen	J554/01	TUB 12438	EU049214	EU054859	–	–
*H. isatidis*	*Isatis tinctoria*	Germany, Sachsen-Anhalt, Rollsdorf	J928/01	TUB 12429	AY531443	EU054851	–	–
*H. lobulariae*	*Lobularia maritima*	Germany, Sachsen-Anhalt, Arendsee	J3454/01	TUB 12414	AY531410	EU054856	–	–
*H. lunariae*	*Lunaria rediviva*	Austria, Niederösterreich, Lilienfeld	HV362	WU22867	AY531401	EU054891	–	–
*H. malyi*	*Cardamine graeca*	Greece, Korfu, Episkepsis	HV2895	WU 32397	KC495022*	KC495022*	KC494943*	KC494982*
*H. nasturtii-aquatici*	*C. amara*	Austria, Oberösterreich, Raab	HV2094	WU 32398	KC495023*	KC495023*	–	KC494983*
*H. nasturtii-aquatici*	*C. amara*	Czech Republic, Krusne Hory, Krystofovy Hamry	D25/5/99	TUB 12448	AY531420	EU054833	–	–
*H. nasturtii-aquatici*	*C. flexuosa*	Germany, Baden-Württemberg, Tübingen, Lustnau	HV834	WU 32399	KC495024*	KC495024*	KC494944*	KC494984*
*H. nasturtii-aquatici*	*C. flexuosa*	Austria, Oberösterreich, St. Willibald	HV2018	WU 32400	KC495025*	KC495025*	KC494945*	KC494985*
*H. nasturtii-aquatici*	*C. flexuosa*	Austria, Tirol, St. Anton am Arlberg		GLM46918	EU049230	EU054895	–	–
*H. nasturtii-aquatici*	*C. flexuosa*	Germany, Sachsen-Anhalt, Koßweda	J121/01	TUB 12434	EU049212	EU054850	–	–
*H. nasturtii-aquatici*	*C. hirsuta*	Austria, Wien, Landstraße, Botanischal Garden	HV2030	WU 32401	KC495026*	KC495026*	KC494946*	KC494986*
*H. nasturtii-aquatici*	*C. hirsuta*	Austria, Oberösterreich, St. Willibald	HV2092	WU 32402	KC495027*	KC495027*	KC494947*	KC494987*
*H. nasturtii-aquatici*	*C. hirsuta*	USA, Maryland, Greenbelt	HV-PA3	WU 32403	KC495028*	KC495028*	KC494948*	KC494988*
*H. nasturtii-aquatici*	*C. hirsuta*	Germany, Sachsen-Anhalt, Tanne	J535/01	TUB 12462	EU049259	EU054919	–	–
*H. nasturtii-aquatici*	*C. hirsuta*	Germany, Nordrhein-Westfalen, Wuppertal	MG1821	TUB 12225	AY531421	AY035505	–	–
*H. nasturtii-aquatici*	*C. pratensis*	Germany, Baden-Württemberg, Tübingen, Lustnau	HV796	WU 32404	KC495029*	KC495029*	KC494949*	KC494989*
*H. nasturtii-aquatici*	*C. pratensis*	Germany, Baden-Württemberg, Tübingen	HV799	WU 32405	KC495030*	KC495030*	KC494950*	KC494990*
*H. nasturtii-aquatici*	*C. pratensis*	Germany, Baden-Württemberg, Tübingen, Lustnau	HV829	WU 32406	–	–	–	KC494991*
*H. nasturtii-aquatici*	*C. pratensis*	Austria, Niederösterreich, Mariensee	HV2400	WU 32407	KC495031*	KC495031*	KC494951*	KC494992*
*H. nasturtii-aquatici*	*C. pratensis*	Germany, Sachsen-Anhalt, Großpörthen	J145/01	TUB 12456	EU049258	EU054918	–	–
*H. nasturtii-aquatici*	*C. pratensis*	Germany, Baden-Württemberg, Tübingen	MG1820	TUB 12221	EU049205	AY035504	–	–
*H. nasturtii-aquatici*	*C. pratensis*	Germany, Baden-Württemberg, Niedernhall	MG1885	TUB 12274	AY531417	EU054825	–	–
*H. nasturtii-aquatici*	*Nasturtium officinale*	Germany, Sachsen-Anhalt, Sülldorf	J3493/01	TUB 12415	AY531419	EU054870	–	–
*H. nasturtii-aquatici*	*N. officinale*	Sweden, Gotland, Visby		GLM50769	EU049233	EU054897	–	–
*H. nesliae*	*Neslia paniculata*	Austria, Niederösterreich, Theresienfeld	HV203	WU22913	AY531458	EU054892	–	–
*H. praecox*	*Draba verna*	Austria, Wien, Botanical Garden	HV2144	WU30285	EU049239	EU054903	–	–
*H. rorippae-islandicae*	*Rorippa palustris*	Austria, Tirol, Steeg		GLM46904	EU049235	EU054899	–	–
*H. sisymbrii-sophiae*	*Descurainia sophia*	Austria, Niederösterreich, Hundsheim	HV276	WU22928	AY531430	EU054910	–	–
*H. teesdaliae*	*Teedalia nudicaulis*	Germany, Sachsen, Zschepa	J1186/01	TUB 12406	AY531415	EU054860	–	–
*H. thlaspeos-arvensis*	*Thlaspi arvense*	Austria, Oberösterreich, St. Willibald	HV762	WU	AY531445	EU054890	–	–
*H. thlaspeos-perfoliati*	*Noccaea caerulescens*	Czech Republic, Krusne Hory, Vejprty	D24/4/99	TUB 12444	AY531433	EU054831	–	–
*H. thlaspeos-perfoliati*	*N. caerulescens*	Germany, Sachsen, Obercunnersdorf	J450/01	TUB 12422	AY531436	EU054857	–	–
*Perofascia lepidii*	*Lepidium ruderale*	Germany, Sachsen-Anhalt, Röden	J2068/01	TUB 12409	AY531467	EU054855	–	–
*Perofascia lepidii*	*L. ruderale*	Germany, Sachsen-Anhalt, Wendelstein	J3189/01	TUB 1241	AY531446	EU054854	–	–
*Peronospora aubrietae*	*Aubrieta* sp.	Sweden, Gotland, Visby		GLM50765	EU049228	EU054893	–	–
*Peronospora crispula*	*Reseda lutea*	Austria, Burgenland, Apetlon	HV1028	WU	AY531437	EU054847	–	–
*Peronospora lepidii-sativi*	*Lepidium draba*	Austria, Niederösterreich, Guntramsdorf	HV246	WU22908	AY531463	EU054889	–	–
*Peronospora rumicis*	*Rumex acetosa*	Austria, Oberösterreich, Kopfing	HV312	WU22925	AY198287	KC495032*	KC494952*	KC494993*

### DNA extraction, PCR and sequencing

For DNA extraction, infected dry host tissue was placed in 2-ml reaction tubes together with six sterile 2-mm glass beads and ground in a Retsch 200 mixer mill for 10 min at a frequency of 30 Hz. DNA was extracted using the modified CTAB protocol described in Riethmüller et al. (2002).

A ca. 2,200-bp-long fragment containing partial nuSSU-ITS-LSU rDNA was amplified using primers DC6 (Bonants et al. 1997) and LR6-O (Riethmüller et al. 2002) or LR6-O1 (designed here; 5′ CGCATCGCCAGACGAGC 3′). In cases where no product could be obtained, ITS and LSU were separately amplified using primers DC6 and ITS4 (White et al. 1990) and LR0R (Vilgalys and Hester 1990) and LR6-O1, respectively. For cycle sequencing, primers ITS5-P (designed here; 5′ GGAAGGTGAAGTCGTAACAAGG 3′), ITS4, LR0R and LR6-O were used. For the mitochondrial cytochrome c oxidase subunit I (*cox1*) sequences, primers Oom-CoxI-lev-up and Oom-CoxI-lev-lo (Robideau et al. 2011) were used for amplification and cycle sequencing; the cytochrome c oxidase subunit II (*cox2*) was amplified and cycle-sequenced with the forward and reverse primers of Hudspeth et al. (2000). The PCR products were purified using an enzymatic PCR cleanup (Werle et al. 1994) according to the protocol of Voglmayr and Jaklitsch (2008). DNA was cycle-sequenced using the ABI PRISM Big Dye Terminator Cycle Sequencing Ready Reaction Kit v.3.1 (Applied Biosystems, Warrington, UK) and an automated DNA sequencer (AB 3730xl Genetic Analyzer, Applied Biosystems).

### Phylogenetic analysis

To reveal the phylogenetic position of the *Hyaloperonospora* clades on *Cardamine*, an ITS-LSU data matrix was used. For this, the new sequences generated during the current study were combined with those of accessions from *Cardamine* and *Nasturtium* included in Göker et al. (2009a); in addition, sequences of representative species from throughout the tree of Göker et al. (2009a) were added (Table 1), with *Perofascia lepidii* as outgroup to root the trees. All alignments were produced with Muscle v.3.6 (Edgar 2004).

For evaluation of species status, a multi-gene analysis of all four genes (ITS, LSU, *cox1*, *cox2*) was performed; due to lack of data available for other species, only accessions of all six clades from *Cardamine* were included, with *Peronospora rumicis* as outgroup to root the tree. Prior to phylogenetic analyses, the approach of Wiens (1998) was applied to test for significant levels of localized incongruence among the two gene partitions, using the level of bootstrap support (Sung et al. 2007). For this, the 70 % maximum parsimony (MP) bootstrap trees of the individual gene regions (ITS-LSU, *cox1*, *cox2*) were compared, which were calculated using the same parameters as for the combined analysis given below. No topological conflicts were observed between these bootstrap trees of genes, indicating the absence of significant incongruence and combinability of the matrices (Wiens 1998).

Maximum parsimony (MP) analysis was performed with PAUP* v.4.0 b10 (Swofford 2002), using 1,000 replicates of heuristic search with random addition of sequences and subsequent TBR branch swapping (MULTREES option in effect, COLLAPSE=MINBRLEN, steepest descent option not in effect), each replicate limited to 1 million rearrangements. All molecular characters were unordered and given equal weight; analyses were performed with gaps treated as missing data. Bootstrap analysis with 1,000 replicates was performed in the same way, but using 5 rounds of random sequence addition and subsequent branch swapping during each bootstrap replicate.

For maximum likelihood (ML) and Bayesian analyses, the well-known general time reversible model (GTR) was selected by Modeltest 3.6 (Posada and Crandall 1998) using the Akaike information criterion for all three genes; with a gamma distribution for the ITS-LSU (GTR+G) and with invariant sites and gamma distribution for the remaining sites for *cox1* and *cox2* (GTR+I+G). In the combined analyses of all gene regions, substitution parameters were estimated separately for each region. For ML analyses, 500 rounds of random addition of sequences as well as 500 fast bootstrap replicates were computed with RAxML (Stamatakis 2006) as implemented in raxmlGUI 0.95 (Silvestro and Michalak 2012) using the GTRGAMMA and GTRCAT substitution models, respectively. For Bayesian analyses using MrBayes v.3.1.2 (Huelsenbeck and Ronquist 2001), three parallel runs of four incrementally heated simultaneous Markov chains were performed over 1 million generations from which every 100th tree was sampled in each run. The first 500 trees were discarded, and a 90 % majority rule consensus of the remaining trees was computed to obtain posterior probabilities. The final matrix was deposited in TreeBASE (http://www.treebase.org) and is available under http://purl.org/phylo/treebase/phylows/study/TB2:S14017.

## Results

**Fig. 1:**
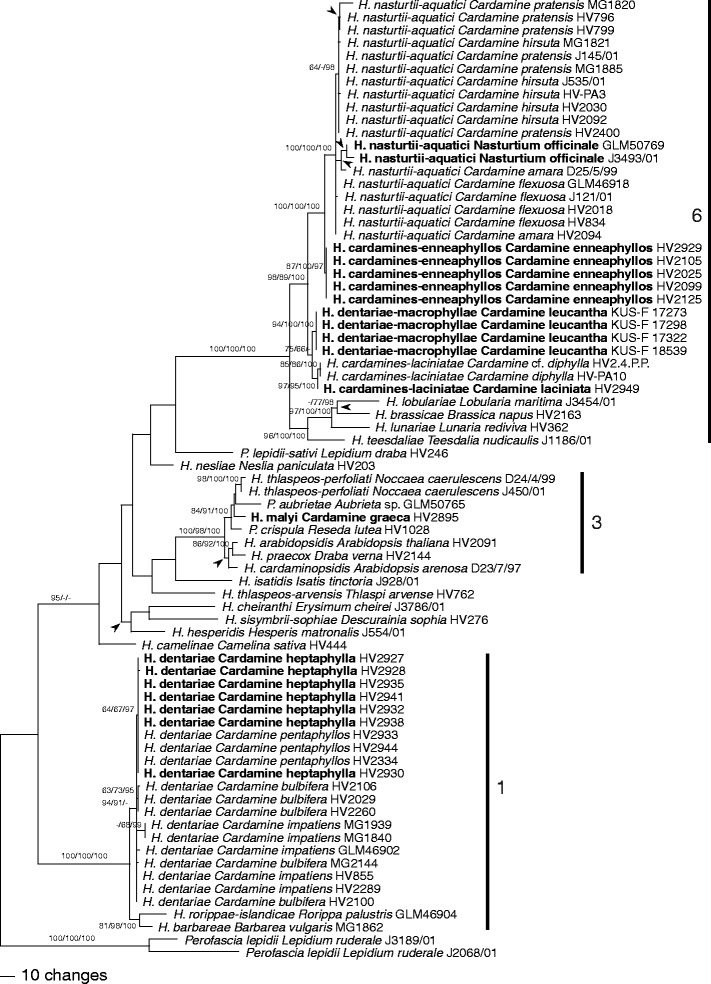
Phylogram showing 1 of 2,106 MP trees inferred from the complete ITS (ITS1, 5.8S rDNA, and ITS2)-LSU alignment with PAUP and rooted with *Perofascia*; *arrowheads* denoting branches/nodes collapsed in the strict consensus tree of all MP trees. MP and ML bootstrap support above 60 % and posterior probabilities above 90 % are given at first, second, and third positions, respectively, above/below the branches. *Hyaloperonospora* specimens from type hosts of the six species from *Cardamine* are shown in *bold*. Clade numbers correspond to those of Göker et al. (2009a)

After the exclusion of excessive leading and trailing gap regions and large insertions present in some single sequences, 2,172 characters were included in the ITS-LSU analyses, of which 428 were parsimony informative. MP analyses revealed 2,106 MP trees of 1,213 steps which were identical except for topologies within species, a polytomy containing *H. lobulariae*, *H. brassicae* and *H. lunariae*, a polytomy containing *H. hesperidis* and the *H. cheiranthi*–*H. sisymbrii-sophiae* clade, and a polytomy containing *H. cardaminopsidis* and the *H. arabidopsidis*–*H. praecox* clade. Backbone topology of the deeper unsupported nodes of the ML tree obtained with RAxML differed from the MP strict consensus tree (data not shown), but topologies of supported nodes were the same as in the MP analysis. One of the 2,106 MP trees is shown as phylogram in Fig. 1, with MP and ML bootstrap support above 60 % and posterior probabilities above 90 % given at first, second and third positions above/below the branches, respectively.

**Fig. 2:**
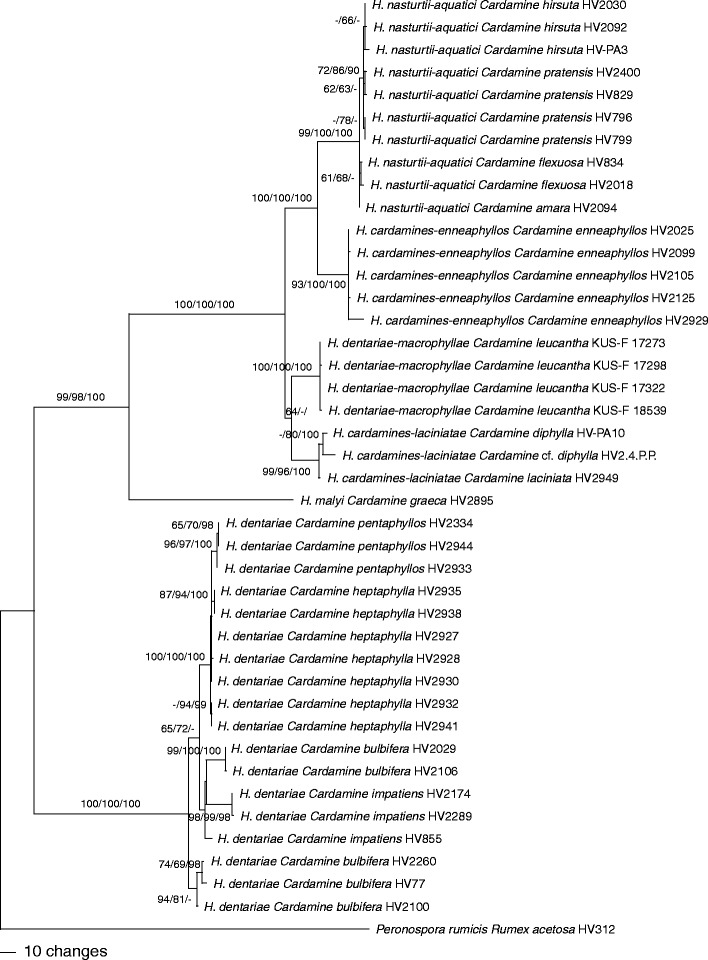
Phylogram showing 1 of 54 MP trees inferred from the combined ITS-LSU-*cox1-cox2* sequence data matrix. MP and ML bootstrap support above 60 % and posterior probabilities above 90 % are given at first, second, and third positions, respectively, above/below the branches

The combined matrix contained 3,412 characters (2,150 from ITS-LSU, 681 from *cox1*, 581 from *cox2*), from which 374 were parsimony informative. MP analyses revealed 54 MP trees of 885 steps which were identical except for topologies within species. Tree topology of the ML tree obtained with RAxML was fully compatible with the MP strict consensus tree (data not shown). One of the 54 MP trees is shown as phylogram in Fig. 2, with MP and ML bootstrap support above 60 % and posterior probabilities above 90 % given at first, second, and third positions above/below the branches, respectively.

In the ITS-LSU analyses (Fig. 1), the accessions from *Cardamine* were contained in three distinct clades of Göker et al. (2009a), one consisting of *H. dentariae* within clade 1, a second containing *H. malyi* within clade 3, and a third highly supported monophyletic clade containing *H. cardamines-laciniatae*, *H. dentariae-macrophyllae*, *H. cardamines-enneaphyllos* and *H. nasturtii-aquatici* within clade 6. In both ITS-LSU and combined analyses, all species were highly supported. Each *Cardamine* species harboured only a single *Hyaloperonospora* species. The accessions from the previously not sampled *C. enneaphyllos* formed a distinct clade representing a new species described as *H. cardamines-enneaphyllos* below. *Hyaloperonospora cardamines-enneaphyllos*, *H. malyi* and *H. dentariae-macrophyllae* each contained only accessions from a single host species, whereas within *H. cardamines-laciniatae*, *H. dentariae* and *H. nasturtii-aquatici* accessions from several host species were placed. Within *H. dentariae* and *H. nasturtii-aquatici*, some substructure was observed in the combined analyses according to the host species, indicating the presence of host-specific lineages within species. Within the *H. dentariae* clade, the accessions from *C. heptaphylla* and *C. pentaphyllos* formed a highly supported monophylum, the latter again forming a highly supported subclade; however, accessions from *C. bulbifera* and *C. impatiens* were not contained in monophyletic lineages. Within *H. nasturtii-aquatici*, the accessions from *C. hirsuta* and from *C. flexuosa* each formed weakly supported subclades, the latter being included in a moderately supported clade together with the accessions from *C. pratensis*.

## Taxonomy

As a result of the molecular phylogenetic investigations, *H. cardamines-enneaphyllos* is described as a new species. In addition, *P. cardamines-laciniatae*, *Peronospora dentariae*, *P. dentariae-macrophyllae*, *P. malyi* and *P. nasturtii-aquatici* are combined into *Hyaloperonospora*.

**Fig. 3:**
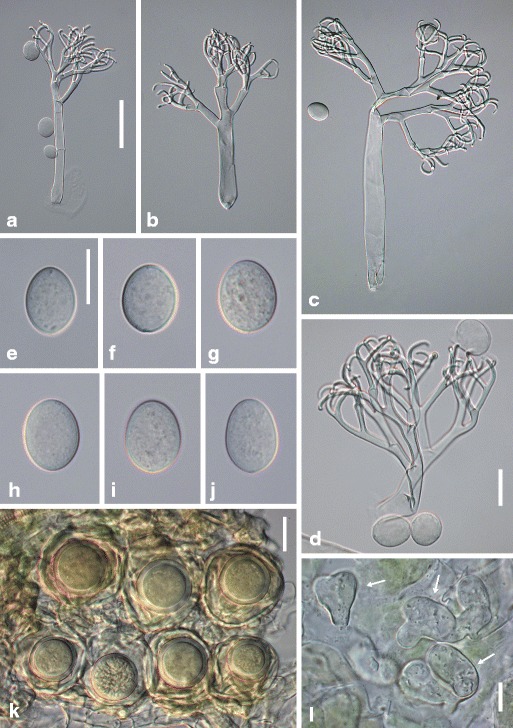
*Hyaloperonospora cardamines-enneaphyllos*. **a**–**c** conidiophores; **d** ultimate branchlets; **e**–**j** conidia; **k** oogonia and oospores in host tissue; **l** three vesicular-lobate haustoria (*arrows*). Sources: (**a**,** d**) WU 32395; (**b**,** c**,** g**–**j**) WU 32393; (**e**,** f**,** k**,** l**) holotype WU 32392.* Scale bars* (**a**–**c**) 50 μm, (**d**–**l**) 20 μm

*Hyaloperonospora cardamines-enneaphyllos* Voglmayr, sp. nov. Fig. 3

Mycobank MB 803659

*Etymology*: Referring to its host, *Cardamine enneaphyllos*.

*Infection* commonly systemic, more rarely localized, when systemic whole plants or leaves stunted, chlorotic, dwarfed, when localized producing polyangular to confluent lesions without distinct margins. *Down* whitish, consisting of scattered conidiophores to dense and felt-like. *Haustoria* lobate, (9−)12−19(−21) μm long, (12−)15–26(−33) μm wide (*n* = 26), filling the host cell partly to almost completely. *Conidiophores* hyaline, stout, straight to slightly sinuous, (135−)200–325(−420) μm long; trunk straight, usually collapsed (flat), (70−)105–200(−290) μm long (*n* = 44), variable in width, 9–17 μm wide below the first branch, 13–25 μm wide above the base, with often somewhat swollen base; callose plugs absent; upper part monopodially or subdichotomously branched 4–5 times. *Ultimate branchlets* in pairs or rarely single, flexuous, sigmoid to strongly involuted, (9−)12−21(−30) μm long, 2–3 μm wide at the base (*n* = 66), apex obtuse. *Conidia* hyaline, subglobose, ellipsoidal to ovate, (17−)20.5–25(−29) μm long, (15−)17–20(−22.5) μm wide, mean 23.3 × 18.0 μm, l/w ratio (1.04−)1.16–1.32(−1.45) (*n* = 215), greatest width median or submedian, base and tip round; pedicel absent in most conidia but a scar visible at the point of attachment; producing germ tubes. *Resting organs* oogonia subglobose to irregular, pale yellowish, (41−)49–59(−64) μm diam., wall smooth, 1.5−4 μm thick (*n* = 43); oospores aplerotic, globose, (25−)28–33(−36) μm diam., wall 2.5–3.5 μm thick (*n* = 43), smooth.

*Habitat*: On living leaves and stems of *Cardamine enneaphyllos* (*Brassicaceae*).

*Holotype*: **Austria**, Niederösterreich, Distr. Mödling, Comm. Gießhübl, deciduous forest ca 700 m SW parking lot, 380 m, map grid 7963/1, 27 Apr. 2003, *H. Voglmayr HV2025*, WU 32392.

*Additional specimens examined*: **Austria**, Niederösterreich, Distr. Mödling, Comm. Gießhübl, deciduous forest ca 700 m SW parking lot, 380 m, map grid 7963/1, 18 Apr. 2004, *H. Voglmayr HV2105*, WU 32394. Distr. Bruck/Leitha, comm. Mannersdorf/Leitha, Schweinsgraben SE Mannersdorf, dediduous forest, 260 m, map grid 8063/3, 17 Apr. 2004, *H. Voglmayr HV2099*, WU 32393. Steiermark, Distr. Liezen, Comm. Gröbming, W Winkl, forest between Stickler and Sticklereck, 1200 m, map grid 8549/1, 10 June 2004, *H. Voglmayr HV2125*, WU 323935. **Italy**, Trentino, SE Storo, Val di Lorina, Val delle Communi, 25 May 2012, *H. Voglmayr & I. Greilhuber HV2929*, WU 32396.

*Comments*: Spore sizes of *H. cardamines-enneaphyllos* correspond to those recorded for the eastern Asian *H. dentariae-macrophyllae* (av. 23.3 × 18.0 μm, Gäumann 1918), under which name accessions from *C. enneaphyllos* have been classified by Săvulescu and Rayss (1930), Lindtner (1957) and Kochman and Majewski (1970). Molecular phylogenies reveal *H. cardamines-enneaphyllos* to be related to but clearly distinct from *H. dentariae-macrophyllae*, which is closest relative of the North American *H. cardamines-laciniatae*. The closest relative of *H. cardamines-enneaphyllos*, *H. nasturtii-aquatici*, differs in smaller spore sizes (av. lengths 17.3–20.5, av. widths 15–17.5; see Table 2).

**Table 2:** Conidial measurements of *Hyaloperonospora* accessions from various *Cardamine* species and from *Nasturtium officinale*

Species	Host	Length range (μm)	Width range (μm)	Mean length (μm)	Mean width (μm)	mean l/w ratio	Reference
*H. cardamines-laciniatae*	***C. laciniata***	(12-)18–22(−28)	(11-)14–18(−21)	19.7	16.2	1.21	Gäumann (1918)
	*C. diphylla*	(18.5-)19.5–24(−29) (*n* = 54)	(16.5-)17.5–20(−22) (*n* = 54)	21.8	18.7	1.16	Present study (WU 32373)
*H. dentariae*	***C. heptaphylla***	(11-)15–19(−23)	(9-)11–14(−18)	16.5	13.4	1.23	Gäumann (1918)
	***C. heptaphylla***	15–21	14–17	–	–	–	Gustavsson (1959)
	***C. heptaphylla***	(15.5-)17–19(−21) (*n* = 55)	(13-)13,5–15,5(−17) (*n* = 55)	18.0	14.5	1.24	Present study (WU 32380)
	*C. bulbifera*	(14.5-)16–19(−21.5) (*n* = 73)	(13-)13.5–15.5(−18) (*n* = 73)	17.6	14.6	1.20	Present study (WU 32376)
	*C. bulbifera*	–	–	19.3	16.0	1.20	Gustavsson (1959)
	*C. impatiens*	(12.5-)14.5–17(−19.5) (*n* = 71)	(11-)12–13.5(−14.5) (*n* = 71)	15.6	12.8	1.22	Present study (WU 32388)
	*C. pentaphyllos*	(15.5-)18–21.5(−24.5) (*n* = 45)	(13.5-)15–17.5(−19.5) (*n* = 45)	19.7	16.2	1.22	Present study (WU 32391)
*H. dentariae-macrophyllae*	***C. leucantha***	(16-)21–26(−29)	(12-)18–21(−24)	23.1	18.8	1.23	Gäumann (1918)
	***C. leucantha***	(19-)22.5–25(−28)	(15-)17–21.5(−25)	23.5	19.1	1.22	Present study (KUS-F 17273)
*H. cardamines-enneaphyllos*	***C. enneaphyllos***	(20.5-)22.5–24.5(−25) (*n* = 32)	(15,5-)16.5–19.5(−21.5) (*n* = 32)	23.3	18.0	1.30	Present study (WU 32392)
	***C. enneaphyllos***	(19-)22–26.5(−29) (*n* = 44)	(16.5-)17.5–21(−22.5) (*n* = 44)	24.2	19.3	1.26	Present study (WU 32393)
	***C. enneaphyllos***	(17-)19.5–22.5(−25.5) (*n* = 73)	(16-)17–19(−20) (*n* = 73)	21.2	17.9	1.18	Present study (WU 32395)
	***C. enneaphyllos***	(19-)20.5–25(−28.5) (*n* = 66)	(15-)16.5–19.5(−22.5) (*n* = 66)	22.7	18.0	1.26	Present study (WU 32396)
	***C. enneaphyllos***	(16-)19–25(−28)	(12-)15–19(−24)	22.0	18.3	1.20	Săvulescu & Rayss (1930)
	***C. enneaphyllos***	–	–	25.0	20.2	1.24	Lindtner (1957)
*H. malyi*	***C. graeca***	(16-)20–28(−33)	(14-)18–22(−25)	26.4	21.2	1.24	Lindtner (1957)
*H. nasturtii-aquatici*	***N. officinale***	ca. 16–27^a^	ca. 13–21^a^	20.3	17.1	1.19	Gäumann (1918)
	***N. officinale***	15–25	14–21	–	–	–	Kochman and Majewski (1970)
	*C. amara*	(15.5-)18–22(−25) (*n* = 86)	(14-)16–19(−22) (*n* = 86)	20.5	17.5	1.12	Present study (WU 32398)
	*C. amara*	–	–	19.1	15.6	1.23	Gustavsson (1959)
	*C. flexuosa*	(14-)16–19(−20) (*n* = 39)	(12.5-)14–16 (−17) (*n* = 39)	17.6	15.0	1.17	Present study (WU 32400)
	*C. hirsuta*	(15-)16.5–19(−21.5) (*n* = 70)	(13-)14–16(−18) (*n* = 70)	17.7	15.2	1.16	Present study (WU 32402)
	*C. hirsuta*	–	–	17.3	15.7	1.10	Gustavsson (1959)
	*C. pratensis*	(15.5-)17.5–20(−21) (*n* = 59)	(14-)15–17(−18) (*n* = 59)	18.8	16.0	1.17	Present study (WU 32406)

Hosts in bold denote type hosts. Note the variability of sporangial sizes commonly observed within collections from the same host, within the same *Hyaloperonospora* species, and the overlap in size range between most *Hyaloperonospora* species

^a^range measurements listed in Gäumann (1918: 528) erroneous; approximate range was estimated from spore size line graphs (Gäumann 1918: 466)

*Hyaloperonospora cardamines-laciniatae* (Gäum.) Voglmayr, comb. nov.

*Basionym*: *Peronospora cardamines-laciniatae* Gäum., Beih. bot. Zbl., Abt. 1 35(1): 523. 1918.

Mycobank MB 803660

Confirmed hosts: *Cardamine laciniata*, *C. diphylla*

Distribution: North America

*Hyaloperonospora dentariae* (Rabenh.) Voglmayr, comb. nov.

*Basionym*: *Peronospora dentariae* Rabenh., Fungi Eur. 86. 1859.

Mycobank MB 803661

Confirmed hosts: *Cardamine heptaphylla*, *C. pentaphyllos*, *C. impatiens*, *C. bulbifera*

Distribution: Europe

*Hyaloperonospora dentariae-macrophyllae* (Gäum.) Voglmayr, Y.J. Chin & H.D. Shin, comb. nov.

*Basionym*: *Peronospora dentariae-macrophyllae* Gäum., Beih. bot. Zbl., Abt. 1 35(1): 523. 1918.

Mycobank MB 803662

Confirmed hosts: *Cardamine leucantha*

Distribution: East Asia

*Hyaloperonospora malyi* (Lindtner) Voglmayr, comb. nov.

*Basionym*: *Peronospora malyi* Lindtner, Glasn. Muz. Srpsk. Zeml. (Bull. Mus. Hist. Nat. Pays Serbe), Ser. B, 9: 141. 1957.

Mycobank MB 803663

Confirmed hosts: *Cardamine graeca*

Distribution: Southern Europe

*Hyaloperonospora nasturtii-aquatici* (Gäum.) Voglmayr, comb. nov.

*Basionym*: *Peronospora nasturtii-aquatici* Gäum., Beih. bot. Zbl., Abt. 1 35(1): 528. 1918.

Mycobank MB 803664

Confirmed hosts: *Nasturtium officinale*, *Cardamine amara*, *C. flexuosa*, *C. hirsuta*, *C. pratensis*

Distribution: Europe, Asia, North America

## Discussion

Phylogenetic analyses of the ITS-LSU data are largely congruent with the results of Göker et al. (2009a) concerning the placement of *Hyaloperonospora* accessions from *Cardamine* (Fig. 1) within their clades 1 and 6. However, there are differences in the deeper unsupported nodes of the tree which may be due to the more extensive taxon and accession sampling of Göker et al. (2009a). This is to be expected, as the deeper nodes of trees inferred from ITS-LSU data mostly lack support. Lack of support for deeper nodes in ITS as well as LSU phylogenies has also been reported from other downy mildew genera like *Peronospora* (e.g., Voglmayr 2003; Choi et al. 2007; García Blázquez et al. 2008; Göker et al. 2009b) or *Plasmopara* (e.g., Voglmayr et al. 2004; Voglmayr and Constantinescu 2008; Voglmayr and Thines 2007).

In the combined analyses, resolution as well as support within the main species clades is improved, showing that *cox1* and *cox2* add substantial resolution to the tree. *cox1*, chosen as barcoding locus for higher animals and considered to be the primary barcoding marker for organisms unless shown to be unsuitable (http://www.barcodeoflife.org), has also been shown to be an appropriate barcoding locus for oomycetes (Robideau et al. 2011), which is confirmed in the current study. *cox2* shows similarly good resolution and may serve as an accessory barcoding marker; it also has some advantages over *cox1*, as it usually amplifies better especially in cases of low DNA quantity or older degraded samples (as also shown in Telle and Thines 2008), and thus *cox2* sequences are available for many more species.

The results of the current study clearly show that the classification of *Hyaloperonospora* accessions from *Cardamine* proposed by Gäumann (1918, 1923) has to be substantially revised. In his classification, accessions from *Cardamine heptaphylla*, *C. impatiens*, *C. amara*, *C. flexuosa*, *C. hirsuta* and *C. pratensis* were placed in *Peronospora dentariae*. However, these are actually contained within two distinct clades in the molecular phylogenies, the first two being included in clade 1 as *H. dentariae*, whereas the latter four are part of clade 6 of Göker et al. (2009a), belonging to *H. nasturtii-aquatici* (Figs. 1, 2). Likewise, his *P. cardamines-laciniatae* contained accessions from *C. bulbifera* and *C. laciniata* which are phylogenetically also contained within clade 1 and 6, respectively, the former being placed in *H. dentariae*. This is understandable, as the conidial sizes and shapes, which were primary criteria for species classification, are similar between most of these phylogenetically distinct entities (Table 2), and other features like conidiophore morphology are highly variable and also unsuitable for morphological distinction (Gäumann 1918, own observations). In addition, conidial sizes show some variability between different collections of the same species (Table 2) and may be dependent on host, environmental conditions, and ontogenetic state of the collection, which has been recently confirmed for *Pseudoperonospora cubensis* (Runge et al. 2012). The inability to distinguish them morphologically led Gustavsson (1959) to synonymize *P. cardamines-laciniatae* with *P. dentariae*. On the other hand, Lindtner (1957) accepted the classification of Gäumann, and distinguished the various species by their deviating mean lengths and widths. Based on conidial sizes, Săvulescu and Rayss (1930) classified accessions from *C. enneaphyllos* under *P. dentariae-macrophyllae*, which was subsequently followed by Lindtner (1957) and Kochman and Majewski (1970).

The data on conidial sizes (Table 2) show that most phylogenetic species occurring on *Cardamine* cannot be distinguished by morphology alone, at least not by characters usually used for species identification; they should be considered cryptic species which is quite common amongst downy mildews (Voglmayr 2008). However, the hosts are diagnostic for the different *Hyaloperonospora* species investigated in the present study. Within *Hyaloperonospora*, usually only a single species is observed on a given host species. In exceptional cases where more than one *Hyaloperonospora* species has been observed to occur on a single host species, they were morphologically quite distinct (Voglmayr and Göker 2011). No such case of overlapping host range has yet been observed in *Hyaloperonospora* on *Cardamine* despite extensive sampling. However, reliable species identification is nowadays only possible by sequence data, especially in groups which are incompletely sampled.

Remarkably, *H. nasturtii-aquatici* contains accessions from two distinct genera, *Cardamine* and *Nasturtium*. Long considered to be closely related to *Rorippa* and sometimes even classified within that genus, it has been shown that *Nasturtium* actually is the closest relative of *Cardamine* (Al-Shehbaz and Price 1998; Franzke et al. 1998). The conidial size recorded for *Hyaloperonospora* accessions from *Nasturtium officinale* (range 15–27 × 13–21 μm, av. 20 × 17 μm; see Table 2) fits well the measurements recorded for accessions from *Cardamine* species placed within *H. nasturtii-aquatici* (range 14-25 × 12.5–22 μm, av. lengths 17.3–20.5 μm, av. widths 15–17.5 μm; see Table 2).

Some phylogenetic substructure was observed within both *H. dentariae* and *H. nasturtii-aquatici*, where subclades were formed according to the host species (Fig. 2), which indicates some host specificity also within species. This is evidence for active evolutionary radiation, which may result in speciation events, and some of these subclades may actually represent taxonomically separable entities on the subspecific level. Therefore, these species may be good candidates for population genetic investigations on a larger scale to investigate host specificity, gene flow, and speciation processes in detail. Remarkably, similar phylogenetic substructures were also observed in *Albugo* on various *Cardamine* species, which either formed phylogenetically distinct species or showed distinct substructures within a species according to their hosts (Ploch et al. 2010). This may imply that a common evolutionary pattern may be present in both *Albugo* and *Hyaloperonospora* on *Cardamine*, and a comparison of their divergence patterns could give some more general insights in the evolution of obligatory parasitic oomycetes in future studies.
